# Late breast implant infection after severe pneumonia – a case report and literature review

**DOI:** 10.1080/23320885.2026.2632528

**Published:** 2026-02-21

**Authors:** Erica Segura, Hector Oyonate, Pablo García Barón, Ferran Escrigas, Jordi Descarrega, Joan Fontdevila

**Affiliations:** Plastic Surgery Department, Hospital Clínic de Barcelona, Barcelona, Spain

**Keywords:** Brest reconstruction, implant infection, sonication, late infection

## Abstract

Late infection associated with breast implants is an uncommon but potentially serious complication in patients undergoing breast reconstruction. These infections are rare, poorly documented and may present many years after the initial surgery. We present the case of a 60-year-old woman with a history of mastectomy and breast reconstruction with an implant, who developed a late implant infection nine years after surgery following a severe pneumonia which required intensive care unit stay and intravenous antibiotherapy regime. Despite multiple antibiotic regimens, the patient required implant removal. Sonication of the explanted implant identified *Staphylococcus epidermidis*, highlighting the diagnostic utility of this method. This case underlines the need to consider late implant infections in patients with inflammatory breast symptoms, even years after reconstruction. Implant sonication improves microbiological diagnosis and surgical removal remains the definitive treatment when medical therapy fails.

## Introduction

Breast reconstruction with implants is a widely used technique in patients who have undergone mastectomy, offering both aesthetic and psychological benefits. Hence, it improves quality of life of those patients intervened [1,2]. Despite advances in surgical techniques and management protocols, infection remains one of the main complications, with reported incidence rates ranging from 1% to 2.5% depending on surgical context and patient risk factors [3,4]. Although early infections are more common, late infections, occurring years after the procedure, are extremely rare and poorly documented. These are often associated with bacteraemia resulting from distant infections or invasive procedures [5].

The pathogenesis of late infections differs from early ones, often involving the formation of bacterial biofilms that complicate treatment [6]. Early identification and appropriate management of these infections are essential to preserve patient health and prevent long-term complications.

The aim of this study is to present a rare case of late breast implant infection diagnosed nine years after reconstruction, to discuss its possible pathophysiological mechanisms, and to highlight the diagnostic role of implant sonication together with the therapeutic relevance of early surgical explantation.

## Case presentation

**Figure 1.**
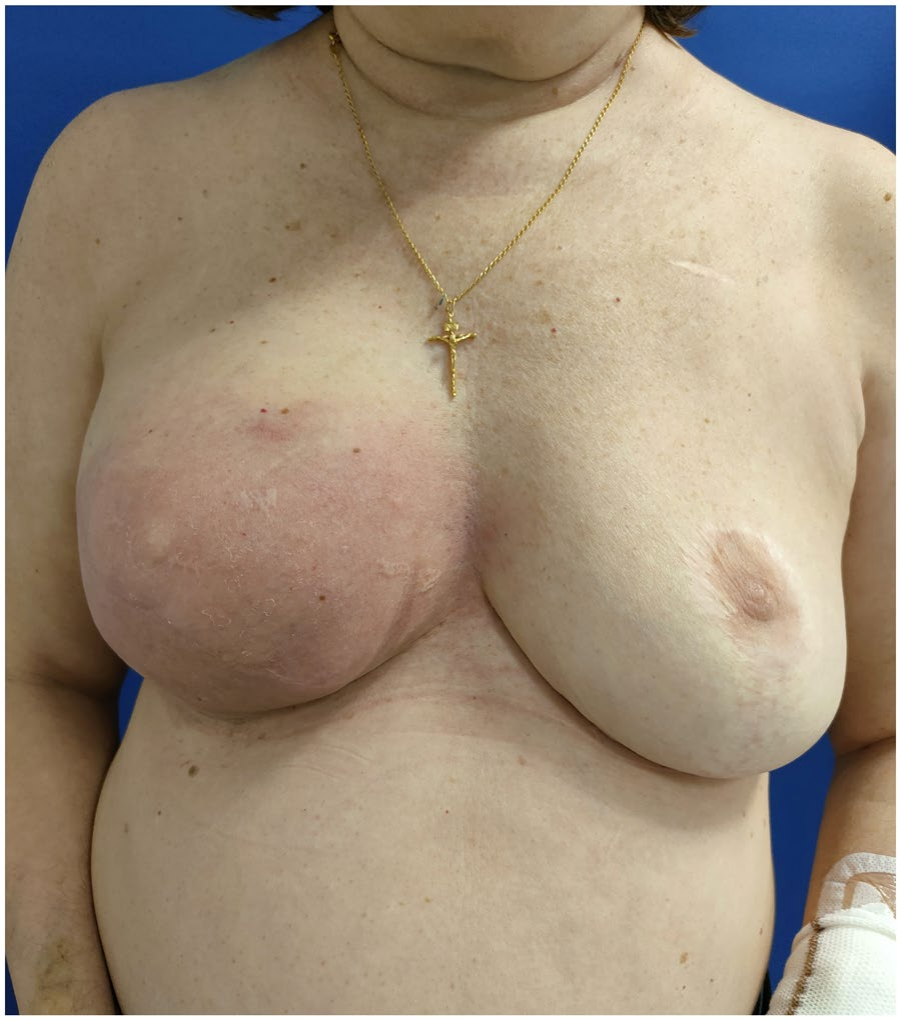
A 60-year-old woman is shown 9 years after right breast reconstruction with silicone gel implant with the presence of erythema.

A 60-year-old woman with a history of right mastectomy for breast cancer in 2012, followed by two-stage prosthetic breast reconstruction in 2015. Nine years later, in 2024, after an uneventful post-reconstruction period, she developed erythema in the lower pole of the right breast, without associated fever (Figure 1). She did not report any trauma or physical injury to the breast. As a recent medical history, one month prior to the onset of breast symptoms, the patient had suffered from *Legionella pneumophila* pneumonia, which required a three-week hospital stay, including admission to the intensive care unit for respiratory failure, and was treated with prolonged intravenous levofloxacin therapy.

**Figure 2.**
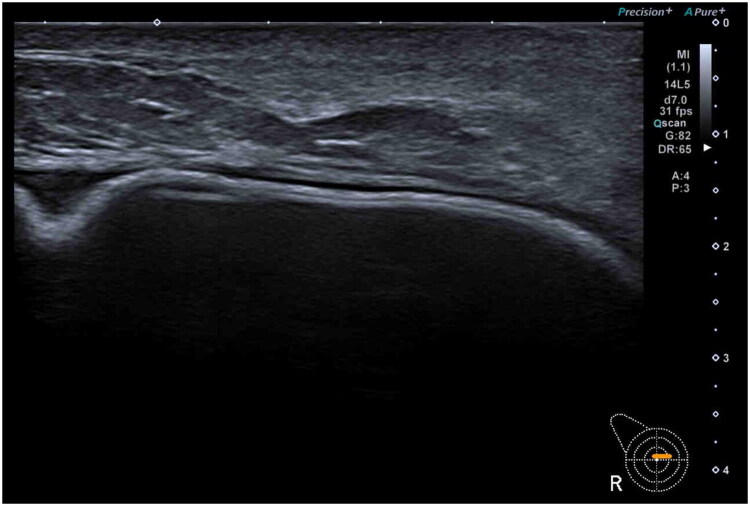
Right breast ultrasound showing a small seroma surrounding the implant. No organized abscess was identified.

Initially, the patient was treated with oral antibiotherapy (amoxicillin–clavulanate) for 10 days. However, the patient did not show signs of improvement so a blood test and an ultrasound were mandated. Protein C-reactive was altered in blood test, reaching a peak of 8 mg/dl without white cell elevation. Breast ultrasound showed no organized abscess but revealed a small seroma surrounding the implant (Figure 2).

**Figure 3.**
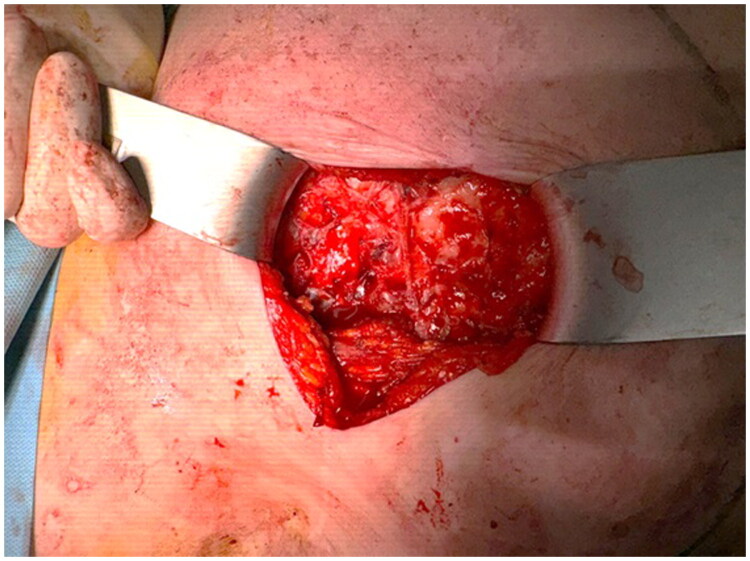
The aspect of the periprosthetic capsule during surgery.

A new antibiotic regime was established (ceftriaxone + clindamycin), but the patient still was not improving so finally surgical explantation was performed. Surgery was uneventful. Intraoperatively, the periprosthetic capsule showed signs of capsular contracture. Samples of the fluid and periprosthetic capsule were taken for microbiological and histopathological analysis (Figure 3), and the implant was sent for sonication testing. The surgical site was irrigated thoroughly with saline and povidone–iodine, a drain was placed, and the wound was closed in layers.

During hospitalization, the patient showed favorable progression, with a gradual reduction in breast cellulitis. The drain had low output and was removed before hospital discharge on the second postoperative day.

Microbiological analysis of the periprosthetic capsule biopsy and the sonicated implant identified *Staphylococcus epidermidis* sensitive to linezolid, rifampicin and vancomycin. Histopathological studies showed no evidence of malignancy. The patient received intravenous antibiotic therapy with linezolid for seven days.

**Figure 4.**
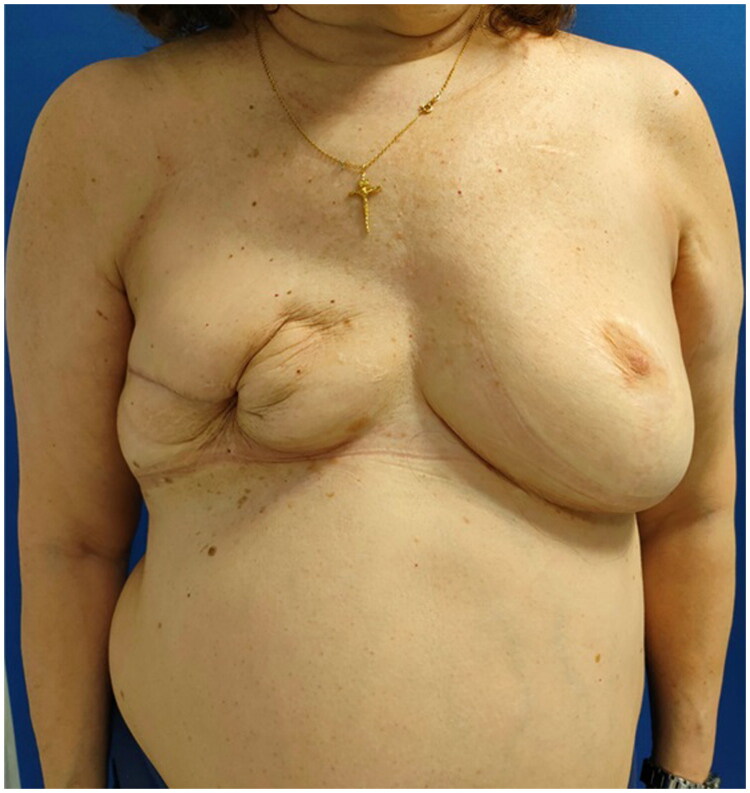
One-month follow-up after surgery explant.

The patient continued follow-up in outpatient clinics with favorable clinical progression, including complete resolution of cellulitis and associated symptoms (Figure 4). Finally, the patient decided not to undergo further breast reconstruction.

## Discussion

Prosthetic infections associated with breast reconstruction, although relatively infrequent, represent one of the main causes of postoperative morbidity in reconstructive breast surgery. Most of these infections occur in the immediate postoperative period, but they can also present later, even years after the initial surgical procedure [7]. This clinical case of late infection by *Staphylococcus epidermidis*, more than a decade after breast implant placement, represents a rare but relevant clinical entity, and provides insight into diagnostic and therapeutic aspects.

In larger studies, overall infection rates in patients undergoing breast reconstruction range between 1% and 2.5%, with lower incidence in cosmetic augmentation procedures [3,8]. Other reports have observed higher rates, particularly in reconstructive surgeries, where infection risk increases in the presence of factors such as smoking, diabetes, radiotherapy and prolonged operative time [4,9]. However, the incidence of late infections – those occurring beyond six months or even years after surgery – is significantly lower, with prevalence under 1% [10].

In this case, the patient had no risk factors at the time of implant placement and remained completely asymptomatic for nearly 10 years. The appearance of local inflammatory symptoms occurred weeks after a severe *Legionella pneumophila* pneumonia that required prolonged hospitalization. Although blood cultures were negative and *S. epidermidis* bacteraemia was not detected, it is plausible that the transient immunosuppression caused by the severe pulmonary infection and intensive care unit stay facilitated the activation of a previously subclinical colonization of the breast implant [11].

Literature indicates that *Staphylococcus epidermidis* is one of the main pathogens involved in chronic or late breast implant infections due to its ability to form biofilms on inert surfaces [12]. These biofilms protect bacteria from both the immune system and antibiotics, explaining the resistance to multiple antimicrobial treatments and the frequent need for surgical implant removal as the only definitive measure.

A key feature of this case was the use of implant sonication, which enabled identification of the responsible pathogen. This technique, which dislodges bacteria adhered to the implant surface, significantly improves diagnostic sensitivity compared to conventional cultures of periprosthetic fluids or tissues. Its utility has been widely supported in the context of orthopedic prosthesis infections and, more recently, for breast implants [13–15].

The diagnostic evaluation of implant-related infections is limited by the ability to recover bacteria embedded in biofilms adherent to the implant surface. Moris et al. compared different biofilm dislodgement techniques for *Staphylococcus epidermidis* on medical implants, including sonication, enzymatic treatment with dithiothreitol, bead milling and combined approaches. Their results showed that sonication was the most effective method for releasing viable bacteria from silicone and other flexible implants, providing significantly higher bacterial recovery than the other techniques tested. These findings support the use of implant sonication in the present case and explain its diagnostic advantage over conventional microbiological cultures [16].

Although hematogenous spread is traditionally considered the main mechanism of late infection in these cases with preceding infectious processes, the mismatch between the microorganism isolated during pneumonia (*Legionella pneumophila*) and that found in the implant (*S. epidermidis*) suggests a different etiopathogenesis [4]. Most likely, there was a subclinical colonization of the implant from the time of placement, later activated by a combination of immunosuppressive factors such as *Legionella* sepsis, broad-spectrum antibiotic use and systemic stress from critical illness.

Studies such as those by Sinha et al. have emphasized that many postoperative infections that occur outside the immediate period may be more related to local skin microbiota than systemic sources [17]. Moreover, research by Washer and Gutowski confirms that late infections, though infrequent, are underreported and may present with atypical clinical features such as localized inflammation without fever or significant systemic inflammatory markers [9].

Finally, it is worth noting that the patient did not present signs of systemic infection or major complications after implant removal and opted not to undergo a new reconstruction. This favorable outcome is consistent with clinical experience suggesting that early explantation in suspected biofilm-associated infections may prevent systemic complications and help preserve surrounding tissue integrity.

## Conclusions

Late breast implant infections, though rare, should be considered in patients presenting with local inflammatory signs – even many years after reconstruction. In this case, temporary immunosuppression following *Legionella* pneumonia may have triggered activation of a subclinical *Staphylococcus epidermidis* colonization, without evidence of hematogenous dissemination.

The reviewed literature suggests that patients with breast implants should be closely monitored following events that may lead to bacteraemia and/or immunosuppression to prevent serious late complications.

Implant removal remains fundamental in cases refractory to medical treatment. This complication highlights the need for long-term surveillance in patients with breast prostheses.
